# CTCF genetic alterations in endometrial carcinoma are pro-tumorigenic

**DOI:** 10.1038/onc.2017.25

**Published:** 2017-03-20

**Authors:** A D Marshall, C G Bailey, K Champ, M Vellozzi, P O'Young, C Metierre, Y Feng, A Thoeng, A M Richards, U Schmitz, M Biro, R Jayasinghe, L Ding, L Anderson, E R Mardis, J E J Rasko

**Affiliations:** 1 Gene and Stem Cell Therapy Program, Centenary Institute, Camperdown, New South Wales, Australia; 2 Sydney Medical School, University of Sydney, Sydney, New South Wales, Australia; 3 Gynaecological Oncology, Royal Prince Alfred Hospital, Camperdown, New South Wales, Australia; 4 Cell Motility and Mechanobiology, School of Medical Sciences, University of New South Wales, Sydney, New South Wales, Australia; 5 Cancer Genomics, McDonnell Genome Institute, Washington University in St Louis, St Louis, MO, USA; 6 Division of Oncology, Department of Medicine, Washington University in St Louis, St Louis, MO, USA; 7 Department of Tissue Pathology and Diagnostic Oncology, Royal Prince Alfred Hospital, Camperdown, New South Wales, Australia; 8 Cell and Molecular Therapies, Royal Prince Alfred Hospital, Sydney, New South Wales, Australia

## Abstract

CTCF is a haploinsufficient tumour suppressor gene with diverse normal functions in genome structure and gene regulation. However the mechanism by which CTCF haploinsufficiency contributes to cancer development is not well understood. CTCF is frequently mutated in endometrial cancer. Here we show that most CTCF mutations effectively result in CTCF haploinsufficiency through nonsense-mediated decay of mutant transcripts, or loss-of-function missense mutation. Conversely, we identified a recurrent CTCF mutation K365T, which alters a DNA binding residue, and acts as a gain-of-function mutation enhancing cell survival. CTCF genetic deletion occurs predominantly in poor prognosis serous subtype tumours, and this genetic deletion is associated with poor overall survival. In addition, we have shown that CTCF haploinsufficiency also occurs in poor prognosis endometrial clear cell carcinomas and has some association with endometrial cancer relapse and metastasis. Using shRNA targeting CTCF to recapitulate CTCF haploinsufficiency, we have identified a novel role for CTCF in the regulation of cellular polarity of endometrial glandular epithelium. Overall, we have identified two novel pro-tumorigenic roles (promoting cell survival and altering cell polarity) for genetic alterations of CTCF in endometrial cancer.

## Introduction

Uterine malignancies represent the most prevalent gynaecological cancers in the developed world. These tumours are becoming an increasing health burden due to associations with increased body mass index, nulliparity and increased life expectancy.^1, 2^ The majority (95%) of these tumours are endometrial carcinomas that originate from the endometrial glandular epithelium. The remaining 5% have a mesenchymal component such as endometrial stromal sarcoma or are mixed epithelial and stromal tumours such as adenosarcomas and carcinosarcomas.^3, 4^ Within endometrial carcinoma, the majority of tumours (~80%) are endometrioid adenocarcinomas, which are generally hormone-responsive, are typified by *PTEN* deletion, and carry a good prognosis. The next most common subtypes are serous carcinoma (5–10%) and clear cell carcinoma (<5%), which are generally non-hormone-responsive and associated with a poorer prognosis. Additionally, serous tumours often carry *TP53* mutations.^3, 4, 5^ Rarer subtypes include mucinous carcinoma, squamous cell carcinoma, undifferentiated carcinoma, and mixed carcinoma which by definition contains at least two histological cell types such as endometrioid and serous carcinoma.^3^

Cancer genome sequencing studies focusing on large patient cohorts of specific cancers are revealing the molecular genetic landscapes of tumours. Recently, we showed that endometrioid tumours had frequent mutations in *PTEN*, *CTNNB1*, *PIK3CA*, *ARID1A*, *ARID5B* and *KRAS*.^5^ We also discovered that *CTCF,* encoding the chromatin organising protein CTCF is mutated in about one quarter of endometrial carcinoma.^5^ This was verified in independent endometrial cancer cohorts.^6, 7^ Importantly, of 127 significantly mutated genes (SMGs) in 12 different cancers, *CTCF* was identified as an SMG in endometrial cancer.^8^ *CTCF* mutations have also been identified at similar or lower frequencies in other tumour types including breast and prostate cancer, Wilms’ tumour and leukaemia.^9, 10, 11, 12^ A CTCF mutation has been identified at relapse in acute lymphoblastic leukaemia^11^ indicating a potential role for CTCF mutation in tumour relapse. Specific mutations in the zinc finger (ZF) domain of CTCF result in reduced or abrogated CTCF DNA binding activity at certain cognate target sites, but not others.^10^

CTCF is an 11-ZF DNA binding protein that has been named the 'master weaver of the genome'^13^ due to its diverse functions in the regulation of chromatin structure and function including: inter- and intra- chromosomal interactions, gene regulation, nucleosome positioning, gene insulation, genetic imprinting, demarcation of lamina associated domains, X-chromosome inactivation, alternative splicing and telomere elongation (reviewed in Marshall *et al.*^14^). We first showed that CTCF can repress cell growth and colony formation suggesting a tumour suppressor function for CTCF.^15^ In addition, CTCF expression also confers a protective effect against apoptosis in various cell lines.^16, 17^ CTCF has been shown to modulate the expression of various cancer-associated gene loci including notable examples: *CMYC*,^18^ *IGF2*,^19^ *TP53*,^20^ *TERT*^21^ and *INK4/ARF*.^22^ Loss of CTCF binding via aberrant DNA methylation of critical control regions can induce epigenetic silencing of tumour suppressor loci or lead to activation of oncogenes.^23, 24^ *Ctcf* heterozygous mice are prone to tumour development in various tissues including the uterus.^25^ This verifies *CTCF*’s role as a haploinsufficient tumour suppressor gene.

Here we show that CTCF mutations identified in primary human endometrial carcinoma predominantly showed loss-of-function phenotypes due to nonsense-mediated decay of mutant transcripts or abrogation of functional activity. In addition, we also identified a recurrent mutation in CTCF, K365T, which showed a gain-of-function phenotype promoting increased cell survival following apoptotic insult. To recapitulate *CTCF* haploinsufficiency in *CTCF* wild-type (WT) cells we used shRNA knockdown of CTCF. We show that reduced CTCF expression reduces the proportion of spheroids showing luminal localisation of apical polarity markers F-Actin and ZO-1 in 3D endometrial spheroid culture, indicating CTCF plays a role in regulating the normal structure of endometrial glands. Furthermore, our copy number variation analysis of primary human endometrial cancers demonstrates that *CTCF* deletion occurs more commonly in tumours with a propensity for relapse or metastasis, and in tumours with clear cell histology. These findings provide further evidence that genetic lesions in *CTCF* promote endometrial carcinogenesis.

## Results

### *CTCF* is mutated in endometrioid adenocarcinomas and related cell lines

**Figure 1:**
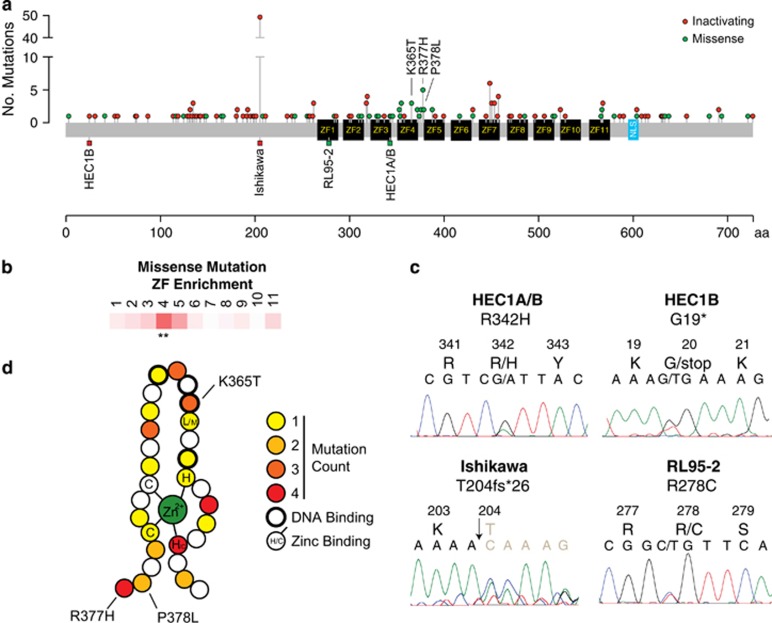
CTCF mutations in human endometrial carcinoma. (**a**) Summary of all *CTCF* inactivating (nonsense and frameshift; red) and missense (green) mutations identified in primary human endometrial carcinomas^5, 6, 7^ (round lollipops, above). Novel *CTCF* mutations identified in endometrial cancer cell lines (square lollipops, below). The location of ZFs (black) and nuclear localisation signal (NLS; blue). Recurrent mutations K365T, R377H and P378L functionally characterised in this study are indicated. (**b**) Heatmap showing the frequency of missense mutations in different ZF domains of CTCF ranging from 0 (white) to 15 (dark pink) residues, **P<0.01 Fisher’s exact test compared to expected. (**c**) Representative electropherograms of somatic mutations in endometrial cancer cell lines: R342H, G19*, T204fs*26, R278C. (**d**) The frequency of CTCF missense mutations found in endometrial carcinoma superimposed on a C2H2 ZF schematic. Note: some ZFs contain only 3 residues juxtaposed between conserved C-terminal histidine residues.

We and others have identified over 200 somatic *CTCF* mutations occurring in primary human endometrial carcinoma samples^5, 6, 7, 26^ (Figure 1a and Supplementary Table 1). The majority (71%) are inactivating mutations resulting from frameshift (45%), nonsense (20%) and splice site mutations (6%). The remaining mutations (29%) are missense mutations with the majority (43/60, 72%, *P*<0.001 Fisher’s exact test) occurring within the 11-ZF DNA binding domain of CTCF. All of the recurrently mutated residues in CTCF (S345, K352, K365, G375, R377, P378, A387, and R567) occur within the ZF domain and are predominantly clustered within ZF 4 (Figure 1b, *P*=0.0041, Fisher’s exact test). As *CTCF* is a significantly mutated gene in endometrial cancer, we sequenced *CTCF* in five endometrial cancer cell lines and identified four mutations (Figures 1a and c). These included two recurrent *CTCF* mutations: T204fs*26 in Ishikawa and R342H in both HEC1A and HEC1B and two novel mutations: G19* in HEC1B and R278C in RL95-2. Interestingly HEC1B, which is a sub-strain of HEC1A isolated from a patient with grade II endometrial cancer,^27^ has acquired an additional somatic mutation in *CTCF*, G19*. Only the KLE endometrial cancer cell line did not contain any *CTCF* mutations. KLE has previously been shown to be diploid for Ch16^28^ and does not contain focal deletions within Ch16q22.1.^29^ The location of missense mutations within the ZF domains of CTCF is shown in Figure 1d and includes mutations affecting zinc ion binding, DNA binding and non-binding residues.

**Figure 2:**
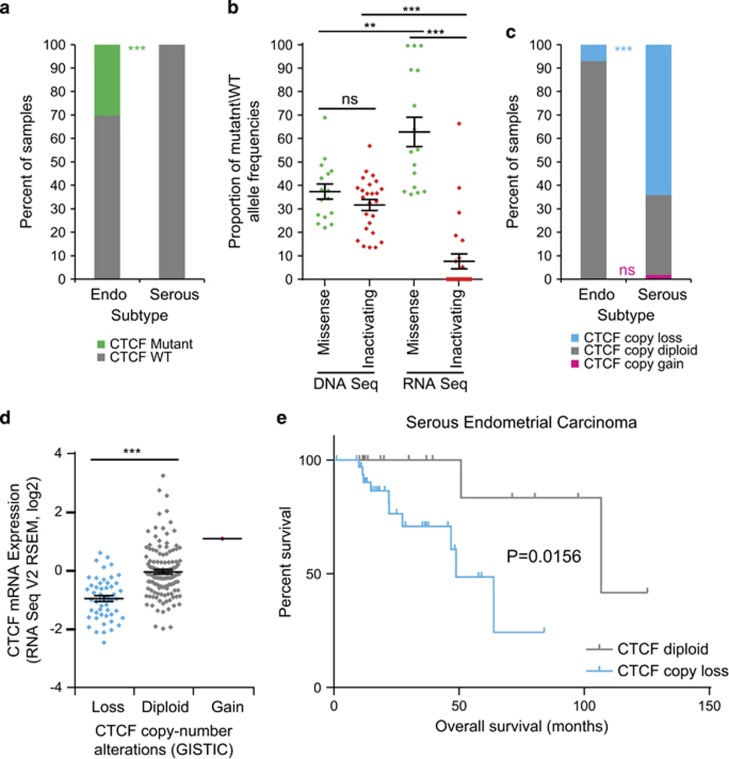
*CTCF* is mutated in endometrioid carcinoma and deleted in serous carcinoma. Analysis of mutation and copy number variation from published data.^5^ (**a**) Proportion of endometrioid (Endo, *n*=172) and serous samples (*n*=60) with mutations in CTCF; ***P<0.001 Fisher’s exact test. (**b**) Proportion of missense (*n*=16) or inactivating (nonsense and frameshift; *n*=25) mutations in all reads obtained by DNA Seq and RNA Seq spanning the mutation site in samples; ns not significant, **P<0.01, ***P<0.001, Mann–Whitney non-parametric test. (**c**) *CTCF* gene copy number analysis of endometrioid (*n*=172) and serous (*n*=60) samples using GISTIC; ***P<0.001 Fisher’s exact test. (**d**) CTCF gene expression in non-CTCF mutant endometrial and serous samples categorised for *CTCF* copy number using GISTIC (Loss *n*=51, Diploid *n*=128 and Gain *n*=1); ***P<0.001 Fisher’s exact test. (**e**) Survival analysis of serous endometrial cancer samples with (*n*=34) or without (*n*=17) *CTCF* genetic deletion; *P*=0.0156, log-rank (Mantel Cox) test.

Within our published patient cohort *CTCF* was sequenced in 172 endometrioid adenocarcinomas and 60 serous carcinomas.^5^ In all, 16 missense and 25 inactivating mutations (including 17 nonsense and 8 frameshift mutations) were identified (Supplementary Table 2). All somatic *CTCF* mutations were identified within the endometrioid subtype (Fisher’s exact test *P*<0.001; Figure 2a). We determined the proportion of mutant to wild-type alleles in tumour samples following DNA and RNA sequencing. In tumour DNA, there was a similar ratio of mutant to wild-type alleles for missense mutations and inactivating mutations (37.6±3.2% and 32.8±2.3%, respectively, mean±s.e.m) consistent with monoallelic mutation of *CTCF* (Figure 2b). The proportion of expressed alleles with missense mutations was 63.1±6.3% and with inactivating mutations was 7.7±3.2% (mean +/- s.e.m.), indicating that most nonsense- and frameshift-mutated alleles were eliminated by nonsense-mediated decay (NMD; Figure 2b). Consistent with nonsense-mediated decay being the mechanism by which nonsense- and frameshift-mutated alleles are degraded, all but two of these mutations obeyed the rule for termination-codon position^30, 31^ in which the premature termination codon must be >55 nt upstream of the last exon-exon junction to result in NMD (Supplementary Table 1). These data indicate that the majority of *CTCF* mutations in endometrial carcinoma result in inactivation of one allele.

### *CTCF* haploinsufficiency occurs in serous endometrial carcinoma

We next determined if somatic copy number alterations in *CTCF* occurred in endometrial carcinoma (as estimated by GISTIC from within the TCGA portal). Loss in *CTCF* copy number occurred in 7.0% of endometrioid and 65.5% of serous subtype tumours, indicating that *CTCF* genetic deletion was more common in poorer prognosis serous subtype tumours (Fisher’s exact test, *P*<0.001; Figure 2c). When samples with somatic *CTCF* mutations were excluded, relative linear copy number values for *CTCF* correlated with CTCF mRNA expression (ρ=0.384, Spearman’s log-rank correlation). Furthermore, samples with somatic *CTCF* deletion expressed significantly less CTCF mRNA (*P*<0.001, Mann–Whitney test; Figure 2d). *CTCF* copy number gain was observed in only a single serous carcinoma sample. Survival analysis restricted to patients with serous tumours showed that tumours with *CTCF* copy number loss resulted in reduced overall survival compared to *CTCF* diploid tumours (Figure 2e and Supplementary Figure 1). These indicate that genetic deletion of *CTCF*, potentially in concert with other deleted genes on chromosome 16q, results in poorer patient outcomes.

### Loss- and gain-of-function CTCF mutations are observed in endometrial cancer

**Figure 3:**
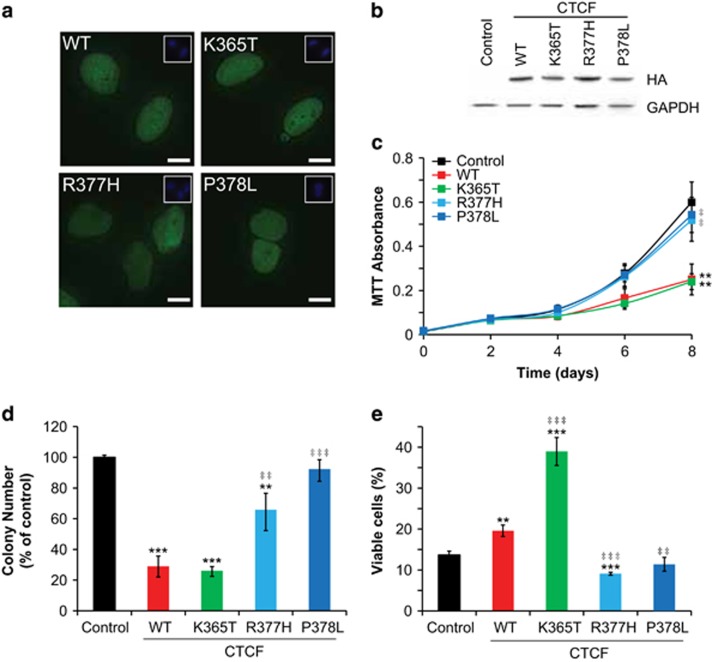
CTCF mutations alter normal CTCF function. (**a**) Subcellular localisation and (**b**) Western blot expression level of HA-tagged wild-type (WT), K365T, R377H and P378L CTCF in Ishikawa cells as detected by the anti-HA antibody; scale bar=10 μm; inset shows DAPI staining of nuclei. Functional analysis of Ishikawa cells expressing CTCF WT, K365T, R377H and P378L, or control eGFP vector: MTT proliferation assay (**c**); colony formation assay (**d**); and apoptosis assay following recovery from UV exposure (**e**); * indicates a significant difference from control; ^‡^ indicates a significant difference from WT CTCF; * or ^‡^*P*<0.05, ** or ^‡‡^*P*<0.01, *** or ^‡‡‡^*P*<0.001 Student’s *T*-test. Data are mean±s.e.m of 3–4 independent experiments.

We incorporated three recurrent CTCF missense mutations found in endometrial carcinoma,^5^ K365T, R377H and P378L (Figures 1a and c) into lentiviral expression constructs containing HA-tagged wild-type (WT) CTCF. We functionally characterised WT and mutant CTCF in *CTCF*-haploinsufficient Ishikawa cells. All three mutants of CTCF were indistinguishable from WT in their nuclear localisation (Figure 3a) and were expressed at similar levels (Figure 3b). We next performed established cellular assays to measure the activity of CTCF including proliferation and colony-forming assays.^15, 17^ Both R377H and P378L abrogated the growth inhibitory effects of WT CTCF (*P*<0.05) such that they were indistinguishable from control eGFP vector (Figure 3c). The K365T mutation did not alter the anti-poliferative effect of WT CTCF. Furthermore, R377H and P378L showed loss-of-function by abrogating the inhibition of clonogenicity of WT CTCF (*P*<0.01 and *P*<0.001 respectively), while K365T again behaved similarly to WT CTCF (Figure 3d). We explored the effect of each mutation on CTCF function further by examining UV-induced apoptosis in Ishikawa cells. WT CTCF has a protective effect compared to control (*P*<0.01; Figure 3e) as expected.^17^ The K365T mutation enhanced this pro-survival effect (*P*<0.001), suggesting a gain-of-function in CTCF while neither R377H nor P378L showed any increase in survival compared to control.

### CTCF knockdown promotes loss of endometrial cell line polarity in 3D culture

**Figure 4:**
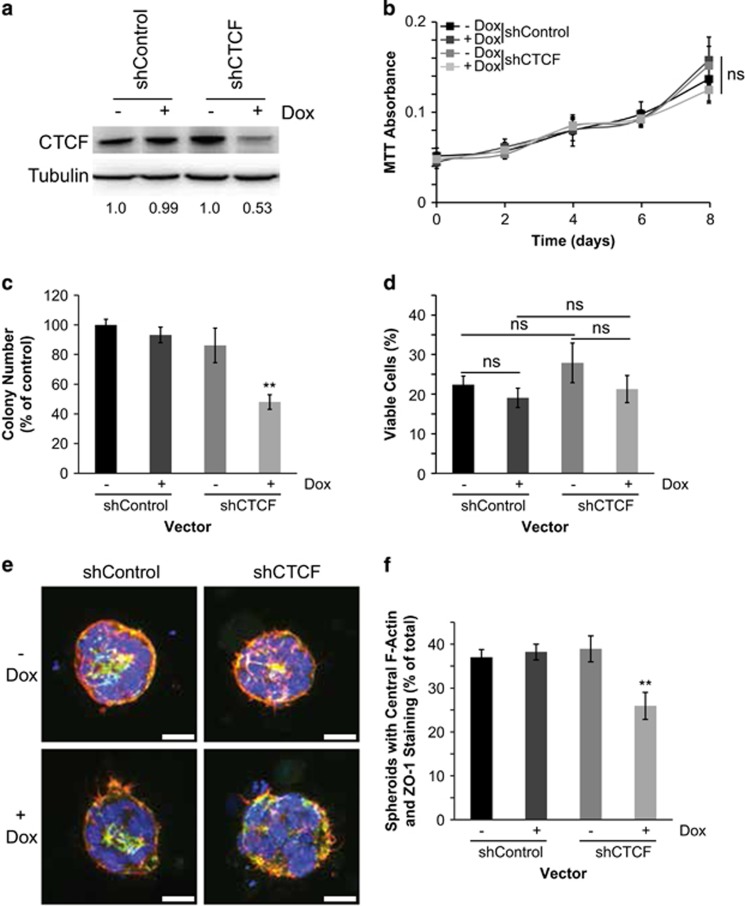
CTCF knockdown alters clonogenicity and endometrial cancer spheroid polarity. (**a**) Immunoblot of KLE cells transduced with inducible shControl or shCTCF shRNA vectors in KLE cells with or without doxycycline (Dox) induction. Densitometric ratio of CTCF expression compared to non-Dox induced samples is shown. Functional analysis of KLE cells expressing either shControl or shCTCF with or without Dox induction: MTT assay (**b**); colony formation assay (**c**); and apoptosis assay following recovery from UV insult (**d**). (**e**) Maximum intensity projections of confocal images of representative KLE endometrial spheroids expressing shControl or shCTCF stained with rabbit anti-ZO-1 (green), F-actin (rhodamine phalloidin, red) and DAPI (blue); scale bar=10 μm. (**f**) Quantitation of spheroids polarisation; ns not significant, **P<0.01 Student’s *t*-test. Data are mean±s.e.m of 3–4 independent experiments.

As the majority of CTCF mutations identified in endometrial carcinoma effectively result in inactivation of one CTCF allele, we modelled this using inducible shRNA knockdown of CTCF. In shCTCF KLE cells we reduced CTCF expression by about half (Figure 4a). However, we did not detect any significant change in cell proliferation after CTCF knockdown (Figure 4b). As with CTCF overexpression studies, we observed a decrease of colony formation with shRNA knockdown of CTCF (Figure 4c). This is consistent with preliminary CRISPR genome editing studies performed in our laboratory (data not shown). However, knockdown of CTCF did not have any effect on KLE cell survival in response to UV treatment (Figure 4d). These data indicate that there is not a simple linear relationship between CTCF expression level and cell characteristics such as cell growth, colony formation and protection from apoptosis. Colony formation in particular shows that neither over- nor underexpression of CTCF is optimal for maximal colony formation.

Endometrial cancer cell lines such as KLE are derived from glandular epithelium, and as such, can be induced *in vitro* to grow spheroids reminiscent of endometrial glands.^32^ These endometrial spheroids display basal/apical polarity when stained for F-actin, a marker of apical polarity based on phalloidin staining, and the apical tight junction protein ZO-1.^33^ Using this staining technique we found that spheroids generated from KLE cells after CTCF knockdown had a reduced proportion of spheroids showing apical/central F-actin and ZO-1 staining compared to control cells (Figures 4e and f). This shows that reduced CTCF gene expression disrupts endometrial cancer cell polarity, a morphological change characteristic of epithelial cancer development.^34^ There was no consistent change observed with CTCF knockdown compared to the three relevant controls in spheroid area, spheroid number, ZO-1 staining intensity or F-actin staining intensity (Supplementary Figure 2).

### CTCF knockdown alters KLE gene expression

**Figure 5:**
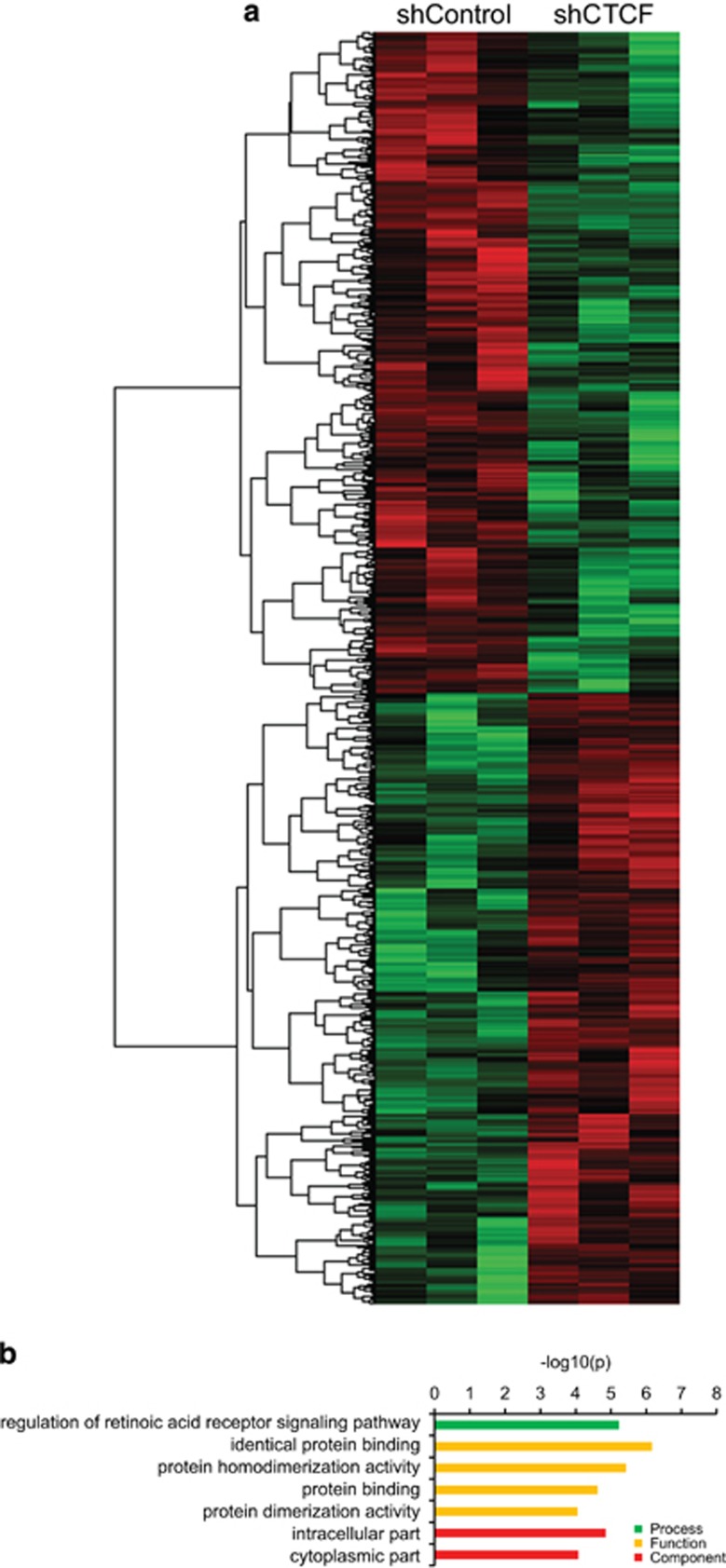
Knockdown of CTCF results in gene deregulation in KLE cells. (**a**) Heat map of significantly (adjusted *P*-value <0.05) differentially expressed genes in shControl (+Dox) samples compared to shCTCF (+Dox) samples. Gene names are provided in Supplementary Table 3. (**b**) Gene ontology analysis of differentially expressed genes showing terms enriched with *P*-value<10^−^^3^ and an FDR *Q*-value of <0.1.

To identify the impact of CTCF knockdown on global gene expression in KLE cells we performed gene expression microarrays. We identified a total of 1744 genes that were differentially expressed in KLE cells after 8 days of induction of CTCF knockdown (Figure 5a and Supplementary Table 3). Gene ontology analysis of this data set revealed significant enrichment for the following terms: Process: regulation of retinoic acid receptor signaling pathway; Function: identical protein binding, protein homodimerisation activity, protein binding, and protein dimerisation activity; Component: intracellular part, cytoplasmic part (Figure 5b).

### *CTCF* genetic deletion is associated with relapse or metastasis, and clear cell histology

**Figure 6:**
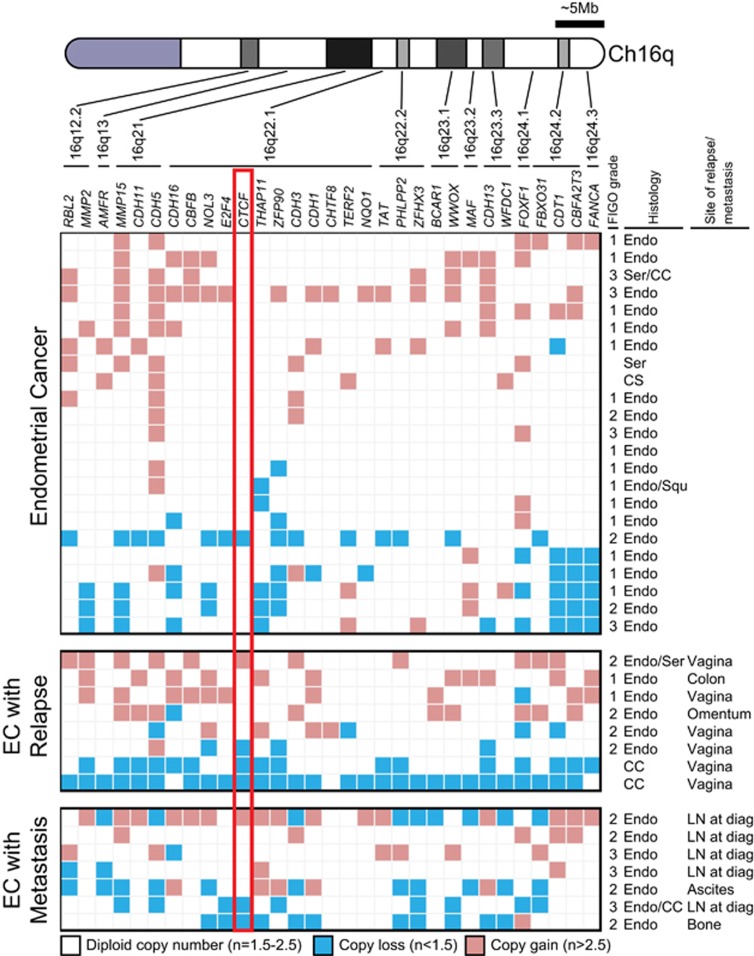
CTCF genetic deletion is associated with relapse and metastasis, and clear cell histology. A custom Nanostring nCounter Copy Number Variation (CNV) Code Set was designed to span cancer-associated genes in the long arm of chromosome 16 with particular focus on genes within 16q22.1 containing the *CTCF* locus (average of three probes, indicated by red border). Chromosome band and gene name are labelled above. An endometrial cancer cohort (*n*=23, top), endometrial carcinomas (EC) which relapsed (*n*=8, middle), and endometrial carcinomas (EC) which metastasised (*n*=7, bottom) were analysed. CNV counts were normalised to the average of seven normal or benign endometrium samples which were all considered diploid and any variation depicted on the heatmap. Federation Internationale de Gynecologie et d'Obstetrique (FIGO) tumour grading scores are shown. Endometrial cancer histologies included endometrioid (Endo), Serous (Ser), Clear Cell (CC), Carcinosarcoma (CS) and Squamous Carcinoma (Squ). Where mixed histologies were seen multiple subtypes are listed. The sites of local relapse or metastasis are listed, and if detected at diagnosis (at diag); (LN) lymph node.

We examined a small independent cohort of 23 primary endometrial malignancies as well as 8 primary tumour samples which developed local relapse and 7 primary tumour samples that presented or later developed metastasis. DNA isolated from FFPE samples was analysed using a custom Nanostring nCounter copy number variation panel of cancer-associated genes across the long arm of chromosome 16 (16q) with a particular focus on 16q22.1 where *CTCF* is located (Figure 6). Within the 23 primary samples only a single grade 2 endometrioid carcinoma showed *CTCF* deletion. Among the 8 tumours that relapsed, 3 showed *CTCF* deletion including a grade 2 endometrioid tumour and two clear cell carcinomas. Among tumours that developed metastases, two of seven showed *CTCF* deletion including a mixed grade 3 endometrioid/clear cell tumour and a grade 2 endometrioid tumour. Collectively *CTCF* was more likely to be deleted in endometrial tumours that developed relapse or metastasis than primary malignancies (*P*=0.0268, Fisher’s exact test). Moreover, two of three relapsed tumours exhibited clear cell histology and one of two metastatic tumours displayed mixed endometrioid/clear cell histology. This indicates a possible association between the presence of clear cell histology and *CTCF* deletion (*P*=0.0259, Fisher’s exact test), which warrants further investigation in a more extensive cohort. We found no evidence that *CTCF* deletion occurred specifically in relapsed or metastatic tumours using matched primary and relapse or metastatic samples (four each, Supplementary figure 3).

## Discussion

We and others^5, 6, 7^ have shown that CTCF is mutated in about one quarter of endometrioid adenocarcinomas. The majority of these somatic *CTCF* mutations are inactivating mutations resulting from nonsense, frameshift or splice site mutations, which we show are rarely expressed due to nonsense-mediated decay^35^ of mutant transcripts, consistent with other reports.^7^ The missense mutations which have been identified occurred predominantly in the 11-ZF DNA binding domain of CTCF. A number of CTCF ZF mutations previously identified in isolated cancers were shown to inhibit CTCF binding to specific DNA binding sites such as *CMYC* and *IGF2/H19* regulatory regions while binding to other sites such as the β-globin and *APPβ* regulatory regions was unaffected.^10^ In endometrial carcinoma these missense mutations appear to cluster within ZFs 4–5 of CTCF. ZFs 4-7 are responsible for binding to the 20 bp core binding motif of CTCF which constitutes ~80% of all CTCF target sites across the genome.^36^

Recurrent CTCF missense mutations in endometrial cancer occur within inter-ZF regions (R377H and P378L), and within conserved residues critical for zinc co-ordination or residues that directly contact the minor groove of DNA (K365T). R377H and P378L are loss-of-function mutations which abrogate the tumour suppressive effects of CTCF. These inter-ZF mutations may alter the conserved DNA binding domain structure and prevent normal CTCF function. Conversely, K365T did not disrupt the tumour suppressive effect of CTCF on cellular growth but enhanced CTCF’s response to UV-induced apoptosis indicating a pro-survival gain-of-function. As this residue directly contacts DNA, this mutation may alter DNA binding specificity. The exact nature or consequences of this altered binding has yet to be determined.

Despite our demonstration of a gain-of-function mutation in CTCF, the majority of CTCF mutations in endometrial cancer result in loss-of-function. We and others have found that the mechanism of *CTCF* gene inactivation differs between cancer subtypes. Microsatellite instability (MSI)-positive endometrioid tumours are prone to strand slippage mutations in *CTCF* resulting in the recurrent T204fs*18 and T204fs*26 mutations.^7^ We found that *CTCF* is genetically deleted in a further 22% of all endometrial carcinomas, and that the highest rates of CTCF genetic deletion occur in endometrial serous carcinomas (65.5%). Consequently, these result in a significant reduction in *CTCF* gene expression, indicating that haploinsufficient deletion of *CTCF* may be important in endometrial carcinoma pathogenesis and/or progression. In addition, other genes within Chr16q are often concurrently deleted with *CTCF*. The haploinsufficienct loss of these additional genes may synergise with *CTCF* haploinsufficiency in promoting more aggressive behaviour in endometrial carcinomas. Thus the mechanism of *CTCF* inactivation could contribute to tumour subtype and prognosis.

*CTCF* has previously been suggested as a target of 16q22.1 deletions observed in various cancer types.^6, 9, 37, 38^ *CTCF* is located within the minimally deleted region of 16q22.1,^37^ however other candidate genes such as E-cadherin (*CDH1*) and *ZFHX3* have also been suggested.^6, 38^ E-cadherin is required for normal development and function of the endometrium,^39^ while loss of E-cadherin staining is associated with endometrial carcinoma development and poor prognosis.^40, 41^ Likewise, somatic *ZFHX3* mutations occur in about 20% of endometrial cancers and are associated with poor patient outcomes.^6^ We have determined that haploinsufficient deletions of 16q, which usually contain *CTCF,* are associated with serous, and now potentially clear cell histology and poorer prognosis. This provides the first evidence that *CTCF* inactivation in conjunction with inactivation of *CDH1* and/or *ZFHX3* and/or other candidate genes on 16q contributes to the development of poorer prognosis tumour subtypes. Any additive or synergistic effect between *CTCF* haploinsufficiency and that of other candidate genes within 16q has yet to be investigated.

CTCF has been established as a tumour suppressor gene due to its ability to suppress cancer cell growth.^15, 17, 25^ Along with *PTEN*, *TP53*, *PIK3CA*, *CTNNB1* and *ARID1A*, *CTCF* has been classified as a significantly mutated gene in endometrial cancer.^8^ *Ctcf* haploinsufficient mice, which are tumour prone, also develop endometrial cancers.^25^ These observations firmly implicate *CTCF* mutation and haploinsufficiency in endometrial carcinoma pathogenesis. However the cellular consequences of *CTCF* mutation and haploinsufficiency had not been investigated. Here we show that at least one recurrent mutation in CTCF, K365T, exhibits a pro-survival gain-of-function phenotype which could contribute to tumourigenesis. To date, this gain-of-function mutation, K365T has been identified in two cases of endometrioid adenocarcinoma^5^ and one serous carcinoma case.^26^ This K365T mutation is the only CTCF mutation to be identified in a worse prognosis serous subtype tumour. Conversely the majority of endometrial cancer-associated genetic changes to CTCF result in inactivation of one allele of *CTCF* leading to haploinsufficiency. Here we show that these can deregulate endometrial cell polarity, a phenotype which is associated with malignancy. Thus, loss-of-function missense mutations, inactivating mutations and genetic copy number loss are different mechanisms which achieve reduced *CTCF* gene dosage and/or expression and/or CTCF protein activity. To date, no homozygous deletion resulting in complete inactivation of CTCF has been reported in tumours. A single case has been reported showing homozygous deletion of *CTCF* exon 3^6^ which encodes the entirety of the N-terminus of CTCF. RNA sequencing was not performed to determine if alternative transcripts of CTCF were expressed. CTCF is absolutely required for development as *Ctcf* null mouse embyros fail to develop into blastocysts.^42^ Thus we expect complete loss of *CTCF* in tumours would be detrimental to cell viability and tumourigenesis, similar to when enforced deletion of both alleles of *DICER1*, a known haploinsufficient tumour suppressor gene, leads to inhibition of tumourigenesis.^43^

*CTCF* haploinsufficiency also destabilises DNA methylation at epigenetically variable CpGs in normal tissues.^25^ It is not clear what specific effects this global epigenetic instability would have on endometrioid cells, but such deregulation may accelerate carcinogenesis, and is indeed recognised as an enabling factor in cancer.^44^ Paradoxically we show that knockdown of CTCF can have both pro-tumourigenic (deregulation of cellular polarity) and anti-tumourigenic (inhibition of clonogenicity) activities. Also, either CTCF overexpression or knockdown inhibit colony formation in endometrial cancer cells. This indicates that CTCF must be tightly regulated to ensure normal cell function.

The endometrium of a reproductive human female (from puberty to menopause, excluding pregnancy) is a dynamic tissue undergoing monthly cycles of proliferation, secretion and menstruation. This cycling is accompanied by dramatic morphological changes in the endometrial glands. However, despite these changes, the glandular epithelial cells maintain normal apical-basal polarity through specific subcellular expression of polarity proteins.^45^ Loss of cellular polarity allows cells to override normal contact inhibition signals and is thus a hallmark of cancer.^44^ This is consistent with the finding of Kemp *et al.*^25^ who showed that CTCF haploinsufficiency did not alter the proliferation of MEFs under subconfluent conditions but showed loss of contact inhibition at confluency. CTCF knockdown in the KLE endometrial cancer cell line reduces the proportion of endometrial cancer spheroids showing central luminal staining of apical polarity markers. Loss of cellular polarity is thought to be an early event in endometrial cancer development, as it is evident in endometrial atypical hyperplasia which progresses to malignant carcinoma in about a third of patients.^46, 47^ Moreover, loss of cellular polarity is an important aspect of epithelial to mesenchymal transition (EMT) known to be involved in tumour progression.^48^ Interestingly, in mice, *Ctcf*^+/^^−^ tumours exhibit increased aggressiveness, including invasion, metastasis and mixed epithelial and mesenchymal morphology.^25^ This is the first time that CTCF has been implicated in the regulation of cellular polarity, and could indicate an important role for CTCF haploinsufficiency in the development of endometrial and other cancers.

Here we showed that knockdown of CTCF resulted in the significant deregulation of gene expression in KLE cells. Using gene process ontology analysis we found significant enrichment for genes associated with the retinoic acid receptor signalling pathway. Interestingly retinoic acid receptor signalling has been associated with differentiation, decidualisation and cancer formation in the endometrium,^49, 50, 51^ and is also associated with the regulation of glandular epithelium polarity.^52^ This may offer a gene regulatory mechanism by which CTCF haploinsufficiency contributes to endometrial cancer development.

In conclusion, we have shown that CTCF mutations and genetic deletions predominantly result in loss-of-function and more rarely pro-survival gain-of-function phenotypes. *CTCF* mutation is restricted to the endometrioid subtype of endometrial carcinoma, while genetic deletion of Ch16q including the *CTCF* locus, occurs predominantly in worse prognosis subtypes including serous carcinoma, and our preliminary studies indicate clear cell carcinoma. Inactivation of one allele of *CTCF* (*CTCF* haploinsufficiency) effectively reduces the amount of functional CTCF in endometrial cancer cells. CTCF knockdown to recapitulate haploinsufficiency resulted in deregulation of endometrial cancer spheroid polarity which likely contributes to endometrial cancer development and progression. Tumours with *CTCF* haploinsufficiency can now be shown to exhibit four different hallmarks of cancer: sustained proliferative signalling, resisting cell death, evading growth suppressors and epigenetic disregulation of the genome.^44^ Our study provides further evidence of CTCF’s role as a haploinsufficient tumour suppressor gene. Future studies to explore the global consequences of *CTCF* haploinsufficiency on three-dimensional genomic architecture and the transcriptional landscape in cancer are now warranted.

## Materials and methods

### Cell culture

Ishikawa, HEC1A, HEC1B, RL95-2 and KLE endometrial cancer cell lines were a kind gift from Associate Professor Deborah Marsh of the Kolling Institute, Sydney. Prior to commencing this study, these cell lines were authenticated by short tandem repeat profiling in 2014 (CellBank, Westmead, NSW, Australia). All cells were cultured in DMEM:F12 medium (Life Technologies, Carlsbad, CA, USA) containing 10% (v/v) FBS and penicillin-streptomycin solution at 37 °C and 5% CO_2_ with or without 1 μg/ml doxycycline (Dox) as indicated. We used established methods for 3D spheroid culture.^32, 33^ Briefly, 96-well imaging plates (Beckton Dickinson, Franklin Lakes, NJ, USA) were coated in Matrigel (Corning, Tewksbury, MA, USA) and allowed to harden. KLE cells (5 × 10^3^/well) were plated in DMEM:F12 medium containing penicillin-streptomycin, 1 mm HEPES, 5 ng/ml EGF, 1:100 dilution of Insulin-Transferrin-Selenium (ITS) supplement (Life Technologies) and 3% (v/v) Matrigel for 10 days with media changes every 2–3 days with or without 1 μg/ml Dox.

### CTCF Sequencing

Genomic DNA was extracted from Ishikawa, HEC1A, HEC1B, RL95-2 and KLE endometrial cancer cell lines using the Purelink genomic DNA isolation kit (Life Technologies). PCR amplicons spanning *CTCF* exons 3-11 including splice sites were amplified with established primers^17^ using Phusion DNA Polymerase (NEB, Ipswich, MA, USA). Each PCR product was Sanger sequenced in forward and reverse directions. All mutations were confirmed by cloning each PCR amplicon into pCR-Blunt-TOPO (Life Technologies) and re-sequencing.

### Generation of lentiviral expression

Point mutations (K365T, R377H and P378L) were introduced into pCCLteteGFP-2A-HA-hCTCF^17^ using splice overlap extension PCR. Sequences for shRNAs targeting human CTCF (shCTCF; 5′-tcccCGAAAGCAGCATTCCTATAttcaagagaTATAGGAATGCTGCTTTCGcttttttc-3′) or encoding an *Arabidopsis thaliana* microRNA 159a (shControl; 5′-tcccTTTGGATTGAAGGGAGCTCttcaagagaGAGCTCCCTTCAATCCAAActtttttc-3′) were cloned into *BbsI/XhoI* sites in the Dox-inducible vector pFH1tUTG.^53^ Lentiviral supernatants for each vector were produced by calcium phosphate transfection of HEK293T cells using Tat-independent packaging plasmids as previously described.^17^ Lentiviral particles were then concentrated by ultracentrifugation for 2 h at 20,000 rpm, 4 °C in a SW28 Beckman rotor. Viral pellets were resuspended on ice in complete Dulbecco's Modified Eagle Medium (DMEM) in 1% of the original volume. Cells were transduced with lentivirus in the presence of Polybrene (8 μg/ml) by spinoculation for 1.5 h. The transduction media on cells was then replaced with fresh media. Approximately 48 h post transduction, GFP-positive cells were sorted on a FACSAria IIu (Becton Dickinson, Franklin Lakes, NJ, USA) and either plated immediately for cell biology assays (CTCF mutants) or cultured in the absence of Dox (shCTCF) until experiments were performed.

### Immunoblotting, immunofluorescence, imaging and quantitation

For Western blotting, cell lysates were electrophoresed on NuPAGE Novex 4-12% Bis Tris Protein Gels (Life Technologies) and transferred to nitrocellulose membrane using the iBlot System (Life Technologies). Membranes were blocked in either 5% (v/v) skim milk in phosphate buffered saline 0.1% (v/v) Tween-20 (PBST) or 1% (w/v) polyethylene glycol MW3350, 1% (w/v) polyvinylpyrrolidone, 0.3% (w/v) bovine serum albumin in PBST for 1 h at room temperature then incubated with 1:1000 mouse anti-HA (Covance, Princeton, NJ, USA), 1:1000 rabbit anti-CTCF^15^ or 1:1000 mouse anti-tubulin (Santa Cruz, Dallas, TX, USA) in blocking buffer. Blots were washed three times each for five minutes in PBST. Membranes were incubated with anti-mouse/rabbit HRP (1:5000) in blocking buffer for 1 h at room temperature. Membranes were the treated with chemiluminescent reagent and exposed on an Image Station 4000R Pro (Kodak, Rochester, NY, USA). Densitometry analysis was performed using Image J software (NIH, Bethesda, MD, USA).

Cells or spheroids were rinsed in PBS and fixed in 10% (v/v) neutral buffered formalin (Fronine, Riverstone, NSW, Australia) for 10 min at room temperature (RT). Samples were washed 3 times for 5 min in phosphate buffered saline (PBS) and then permeabilised with 0.5% (v/v) Triton X-100 in PBS for 10 min at RT. Samples were then washed in PBS as above. Samples were blocked in 20% (v/v) BlokHen (Aves Labs, Tigard, OR, USA) in PBS, 0.02% (v/v) Triton X-100 for 30 min at room temperature and incubated with primary antibodies as follows: mouse anti-HA (1:1,000; Covance) or rabbit anti-ZO-1 (1:100; Cell Signaling, Boston, MA, USA) in blocking buffer for 1.5 h at RT. Samples were then washed 3 times for 5 min in PBS 0.1% (v/v) Triton X-100. Secondary antibodies: anti-mouse Alexa Fluor 594 (1:5,000; Life Technologies) or anti-rabbit Alexa Fluor 488 (1:5,000; Life Technologies) and rhodamine phalloidin to bind F-actin (1:200, Life Technologies) were incubated in blocking buffer for 1 h at RT. Samples were washed 3 times for 5 min in PBS 0.1% Triton X-100. For spheroids the first wash contained 0.2 μg/ml DAPI (Life Technologies) to counterstain nuclei. Cells were mounted in Prolong Gold plus DAPI (Life Technologies), and spheroids were covered with PBS. Imaging of cells was performed on the DeltaVision Personal Deconvolution Microscope (GE Healthcare, Chicago, IL, USA) and images deconvolved using SoftWoRx software (GE Healthcare). Imaging of spheroids for scoring was performed on a BD pathway (Becton Dickinson) and representative images were taken on a Confocal SP5 (Leica, Wetzlar, Germany). Spheroids were scored for central staining of both F-Actin and ZO-1 by a researcher blinded to sample identity. There were an average of 105±6 (mean±s.e.m) spheroids per image, one image per well, and duplicate wells for each experiment. Four replicate experiments were analysed.

### Cell biology assays

All cell biology assays were performed on GFP-positive cells as previously described.^17^ Briefly, MTT cell proliferation assays were performed using 200-250 cells/well plated in a 96-well plate in triplicate and proliferation was assessed at 0, 2, 4, 6 and 8 d by the addition of 3-(4,5-Dimethylthiazol-2-yl)−2,5-diphenyltetrazolium bromide (MTT, Merck, Kenilworth, NJ, USA) overnight. The reaction was quenched with isopropanol/HCl and the optical density at 570 and 630 nm measured using a POLARstar Omega plate reader (BMG Labtech, Ortenberg, Germany). Clonogenicity assays were performed using 1000 cells/10 cm dish in triplicate and incubated for 10-20 d. Cells were rinsed in PBS and fixed with ice-cold methanol, then stained with Giemsa solution diluted 1:20 in H_2_O. The colonies were scored by a researcher blinded to sample identity. For apoptosis experiments, 1 × 10^5^ cells were plated in 24-well plates and incubated overnight. The medium was replaced with PBS and cells were UV irradiated (Ishikawa 1000 μJ, KLE 4000 μJ) in a Stratalinker (Stratagene, San Diego, CA, USA). After 18 h recovery, both adherent and non-adherent cells were stained for flow cytometry using Annexin V-APC (Becton Dickinson) and DAPI (Sigma, Croydon, UK). Viable cells were defined as those cells that were negative for both stains.

### Gene expression array analysis

Triplicate samples of KLE cells expressing either shControl or shCTCF knockdown lentivectors were treated with doxycycline for 8 days prior to isolation of RNA. Gene expression analysis was performed using the GeneChIP PrimeView Human Gene Expression Array (Affymetrix, Santa Clara, CA, USA). Analysis was performed using the Bioconductor packages affy and limma. Array data were normalised using the Robust Multi-array Average (RMA) approach. We applied an intensity based filter to remove probe sets that were not expressed. Differentially expressed genes were identified by fitting a linear model to the data and applying an empirical Bayes smoothing to the standard errors. *P*-values were Benjamini-Hochberg adjusted. Gene ontology analysis was performed using GOrilla (http://cbl-gorilla.cs.technion.ac.il)^54^ using a background of expressed genes, a *P*-value threshold of <10^−3^, and FDR *Q*-value threshold of <0.1.

### Genetic analysis of primary human samples

Archived formalin-fixed, paraffin-embedded (FFPE) primary human endometrial cancer samples and associated clinical data (which was extracted from patient case notes and the secure password-protected hospital database) were deidentified and obtained from the Royal Prince Alfred Hospital in accordance with Sydney Local Health District-approved human ethics protocol (X12-0380). DNA from samples was isolated using the QIAamp DNA FFPE Tissue Kit (Qiagen, Hilden, Germany) according to the manufacturer’s instructions. DNA concentrations were determined by spectrophotometry (Nanodrop) and 500 ng of DNA was prepared for use on a custom nCounter Copy Number Variation Panel (Nanostring, Seattle, WA, USA) with probes measuring the copy number of cancer-associated genes along the long arm of chromosome 16. Copy number values were normalised to seven normal or benign endometrium FFPE samples isolated from the same location which were considered diploid.

## Abbreviations

CTCF: CCCTC-binding factor
Dox: Doxycycline
FBS: Fetal bovine serum
FFPE: Formalin-fixed paraffin-embedded
HRP: Horseradish peroxidase
SMG: Significantly mutated gene
WT: Wild type
ZF: Zinc finger.
