# Supplementary material for: CTCF genetic alterations in endometrial carcinoma are pro-tumorigenic
Source: Oncogene. 2017 Mar 20;36(29):4100–10. doi: 10.1038/onc.2017.25 (PMC5519450; doi:10.1038/onc.2017.25)
Supplement: Supplementary Information [file onc201725x1.docx]

| **Supplementary Table 1:** Mutations in CTCF identified to date in endometrial cancers. To determine if nonsense and frame shift mutations are subject to NMD we determined if mutant transcripts obeyed the >55nt from the 3’ exon-exon junction rule for termination-codon position (Nagy and Maquat (1998). Trends in Biochemical Science. 23(6):198-199) leading to nonsense mediated decay of transcripts. | | | | | | |
| --- | --- | --- | --- | --- | --- | --- |
| **Region** | **CTCF** | **Observed Cases** | **Class of mutation** | **Predicted Function** | **Source** | **PTC position from 3’ exon-exon junction** |
| N-terminus | E2G | 1 | Missense | ? | [^15^](#_ENREF_1) |  |
| N-terminus | G19fs*43 | 1 | Frame shift | Null | [^15^](#_ENREF_1) | >55nt (NMD) |
| N-terminus | G19* | 1 | Nonsense | Null | This study |  |
| N-terminus | K23fs*38 | 1 | Frame shift | Null | [^42^](#_ENREF_2) | >55nt (NMD) |
| N-terminus | R29W | 1 | Missense | ? | [^42^](#_ENREF_2)^,^ [^47^](#_ENREF_3) |  |
| N-terminus | V50fs*12 | 1 | Frame shift | Null | [^47^](#_ENREF_3) | >55nt (NMD) |
| N-terminus | D53fs*24 | 1 | Frame shift | Null | [^42^](#_ENREF_2) | >55nt (NMD) |
| N-terminus | Q72* | 1 | Nonsense | Null | [^15^](#_ENREF_1) | >55nt (NMD) |
| N-terminus | M73fs*5 | 1 | Frame shift | Null | [^42^](#_ENREF_2) | >55nt (NMD) |
| N-terminus | E85* | 1 | Nonsense | Null | [^42^](#_ENREF_2) | >55nt (NMD) |
| N-terminus | E112* | 1 | Nonsense | Null | [^15^](#_ENREF_1)^,^ [^42^](#_ENREF_2) | >55nt (NMD) |
| N-terminus | L115R | 1 | Missense | ? | [^42^](#_ENREF_2) |  |
| N-terminus | Q117* | 1 | Nonsense | Null | [^42^](#_ENREF_2) | >55nt (NMD) |
| N-terminus | T123A | 1 | Missense | ? | [^15^](#_ENREF_1) |  |
| N-terminus | A127fs*4 | 1 | Frame shift | Null | [^15^](#_ENREF_1) | >55nt (NMD) |
| N-terminus | T129fs*3 | 1 | Frame shift | Null | [^42^](#_ENREF_2) | >55nt (NMD) |
| N-terminus | S130* | 2 | Nonsense | Null | [^42^](#_ENREF_2) |  |
| N-terminus | E132fs*24 | 1 | Frame shift | Null | [^42^](#_ENREF_2) | >55nt (NMD) |
| N-terminus | E133* | 3 | Nonsense | Null | [^15^](#_ENREF_1)^,^ [^42^](#_ENREF_2) | >55nt (NMD) |
| N-terminus | Q135fs*19 | 1 | Frame shift | Null | [^42^](#_ENREF_2) | >55nt (NMD) |
| N-terminus | A137fs*17 | 1 | Frame shift | Null | [^42^](#_ENREF_2)^,^ [^47^](#_ENREF_3) | >55nt (NMD) |
| N-terminus | N140fs*2 | 1 | Frame shift | Null | [^42^](#_ENREF_2) | >55nt (NMD) |
| N-terminus | E141fs*12 | 1 | Frame shift | Null | [^47^](#_ENREF_3) | >55nt (NMD) |
| N-terminus | E145* | 1 | Nonsense | Null | [^15^](#_ENREF_1) | >55nt (NMD) |
| N-terminus | L147F | 1 | Missense | ? | [^42^](#_ENREF_2) |  |
| N-terminus | L158fs*21 | 1 | Frame shift | Null | [^42^](#_ENREF_2) | >55nt (NMD) |
| N-terminus | G163E | 1 | Missense | ? | [^15^](#_ENREF_1) |  |
| N-terminus | Q165H | 1 | Missense | ? | [^42^](#_ENREF_2) |  |
| N-terminus | E179* | 1 | Nonsense | Null | [^15^](#_ENREF_1) | >55nt (NMD) |
| N-terminus | Q180* | 2 | Nonsense | Null | [^15^](#_ENREF_1) | >55nt (NMD) |
| N-terminus | Q186* | 1 | Nonsense | Null | [^15^](#_ENREF_1) | >55nt (NMD) |
| N-terminus | S190fs*27 | 1 | Frame shift | Null | [^42^](#_ENREF_2) | >55nt (NMD) |
| N-terminus | W191* | 2 | Nonsense | Null | [^42^](#_ENREF_2) | >55nt (NMD) |
| N-terminus | D194fs*36 | 1 | Frame shift | Null | [^42^](#_ENREF_2) | >55nt (NMD) |
| N-terminus | Y197* | 1 | Nonsense | Null | [^15^](#_ENREF_1) | >55nt (NMD) |
| N-terminus | Q198* | 1 | Nonsense | Null | [^15^](#_ENREF_1) | >55nt (NMD) |
| N-terminus | P200fs*31 | 1 | Frame shift | Null | [^42^](#_ENREF_2) |  |
| N-terminus | T204fs*26,  T204fs*18,  T204fs*19 | 50 | Frame shift | Null | This study [^42^](#_ENREF_2)^,^ [^47^](#_ENREF_3) | >55nt (NMD) |
| N-terminus | T207P | 1 | Missense | ? | [^15^](#_ENREF_1) |  |
| N-terminus | S210fs*19 | 1 | Frame shift | Null | [^42^](#_ENREF_2)^,^ [^47^](#_ENREF_3) | >55nt (NMD) |
| N-terminus | Q233* | 1 | Nonsense | Null | [^42^](#_ENREF_2) | >55nt (NMD) |
| N-terminus | S238* | 1 | Nonsense | Null | [^42^](#_ENREF_2)^,^ [^47^](#_ENREF_3) | >55nt (NMD) |
| N-terminus | N241S | 1 | Missense | ? | [^42^](#_ENREF_2) |  |
| N-terminus | P254S | 1 | Missense | ? | [^42^](#_ENREF_2) |  |
| N-terminus | K256Q | 1 | Missense | ? | [^42^](#_ENREF_2) |  |
| N-terminus | I257fs*11 | 1 | Frame shift | Null | [^15^](#_ENREF_1) | >55nt (NMD) |
| N-terminus | K260fs*2 | 1 | Frame shift | Null | [^42^](#_ENREF_2) | >55nt (NMD) |
| N-terminus | G261_splice | 3 | Splice site | Null | [^15^](#_ENREF_1)^,^ [^42^](#_ENREF_2) | >55nt (NMD) |
| ZF1 | R278C | 1 | Missense | ΔZF structure | This study |  |
| ZF1 | R283H | 1 | Missense | ΔZF structure | [^42^](#_ENREF_2) |  |
| ZF1 | M285fs*25 | 1 | Frame shift | Null | [^47^](#_ENREF_3) | >55nt (NMD) |
| ZF2 | C299fs*15 | 1 | Frame shift | Null | [^15^](#_ENREF_1) | >55nt (NMD) |
| ZF2 | R301fs*13 | 1 | Frame shift | Null | [^42^](#_ENREF_2) | >55nt (NMD) |
| ZF2 | L309M | 1 | Missense | ΔZF structure | [^42^](#_ENREF_2) |  |
| ZF2 | H312R | 1 | Missense | ΔZF structure | [^15^](#_ENREF_1) |  |
| ZF2 | T317fs*91 | 3 | Frame shift | Null | [^15-47^](#_ENREF_1) | >55nt (NMD) |
| ZF2 | G318fs*16 | 2 | Frame shift | Null | [^15-47^](#_ENREF_1) | >55nt (NMD) |
| ZF2 | G318_splice | 2 | Splice site | Null | [^42^](#_ENREF_2) | >55nt (NMD) |
| ZF2 | D328N | 1 | Missense | ΔZF structure | [^42^](#_ENREF_2) |  |
| ZF3 | A330S | 1 | Missense | ΔZF structure | [^42^](#_ENREF_2) |  |
| ZF3 | E336* | 1 | Nonsense | Null | [^42^](#_ENREF_2) | >55nt (NMD) |
| ZF3 | R342H | 2 | Missense | ΔZF structure | This study, [^47^](#_ENREF_3) |  |
| ZF3 | Y343H | 1 | Missense | ΔZF structure | [^15^](#_ENREF_1) |  |
| ZF3 | H345N | 1 | Missense | ΔZF structure | [^42^](#_ENREF_2) |  |
| ZF4 | K352N | 2 | Missense | ΔZF structure | [^42^](#_ENREF_2)^,^ [^47^](#_ENREF_3) |  |
| ZF4 | C353W | 1 | Missense | ΔZF structure | [^42^](#_ENREF_2) |  |
| ZF4 | S354F | 3 | Missense | ΔZF structure | [^15^](#_ENREF_1)^,^ [^42^](#_ENREF_2) |  |
| ZF4 | Y358fs*51 | 1 | Frame shift | Null | [^15^](#_ENREF_1) | >55nt (NMD) |
| ZF4 | K365T | 2 | Missense | ΔZF structure | [^15^](#_ENREF_1) |  |
| ZF4 | K365I | 1 | Missense | ΔZF structure | [^42^](#_ENREF_2) |  |
| ZF4 | R371C | 1 | Missense | ΔZF structure | [^42^](#_ENREF_2) |  |
| ZF4 | R371H | 1 | Missense | ΔZF structure | [^42^](#_ENREF_2) |  |
| ZF4 | H373L | 1 | Missense | ΔZF structure | [^42^](#_ENREF_2)^,^ [^47^](#_ENREF_3) |  |
| ZF4 | G375V | 2 | Missense | ΔZF structure | [^42^](#_ENREF_2) |  |
| ZF5 | R377C | 2 | Missense | ΔZF structure | [^15^](#_ENREF_1) |  |
| ZF5 | R377H | 3 | Missense | ΔZF structure | [^15^](#_ENREF_1)^,^ [^42^](#_ENREF_2) |  |
| ZF5 | P378L | 2 | Missense | ΔZF structure | [^15^](#_ENREF_1)^,^ [^42^](#_ENREF_2) |  |
| ZF5 | A387V | 2 | Missense | ΔZF structure | [^42^](#_ENREF_2) |  |
| ZF5 | D390fs*6 | 1 | Frame shift | Null | [^15^](#_ENREF_1) | >55nt (NMD) |
| ZF5 | R399* | 1 | Nonsense | Null | [^42^](#_ENREF_2) |  |
| ZF6 | F416L | 1 | Missense | ΔZF structure | [^42^](#_ENREF_2)^,^ [^47^](#_ENREF_3) |  |
| ZF6 | H430fs*17 | 1 | Frame shift | Null | [^42^](#_ENREF_2) | >55nt (NMD) |
| ZF6 | H430R | 1 | Missense | ΔZF structure | [^42^](#_ENREF_2) |  |
| ZF7 | T444fs*7 | 1 | Frame shift | Null | [^42^](#_ENREF_2) | >55nt (NMD) |
| ZF7 | R448* | 6 | Nonsense | Null | [^15^](#_ENREF_1)^,^ [^47^](#_ENREF_3) | >55nt (NMD) |
| ZF7 | S450fs*2,  S450fs*61 | 3 | Frame shift | Null | [^42^](#_ENREF_2) | >55nt (NMD) |
| ZF7 | G453_splice | 3 | Splice site | Null | [^42^](#_ENREF_2) | >55nt (NMD) |
| ZF7 | L456* | 1 | Nonsense | Null | [^42^](#_ENREF_2) | >55nt (NMD) |
| ZF7 | R457* | 4 | Nonsense | Null | [^42^](#_ENREF_2)^,^ [^47^](#_ENREF_3) | >55nt (NMD) |
| ZF8 | C469* | 1 | Nonsense | Null | [^42^](#_ENREF_2)^,^ [^47^](#_ENREF_3) | >55nt (NMD) |
| ZF8 | Y471fs*40 | 1 | Frame shift | Null | [^42^](#_ENREF_2) | >55nt (NMD) |
| ZF8 | Y480fs*31 | 1 | Frame shift | Null | [^42^](#_ENREF_2)^,^ [^47^](#_ENREF_3) | >55nt (NMD) |
| ZF8 | H485R | 1 | Missense | ΔZF structure | COSMIC |  |
| ZF9 | K496* | 1 | Nonsense | Null | [^42^](#_ENREF_2) | >55nt (NMD) |
| ZF9 | Q506* | 1 | Nonsense | Null | [^15^](#_ENREF_1) | >55nt (NMD) |
| ZF9 | R515H | 1 | Missense | ΔZF structure | [^15^](#_ENREF_1) |  |
| ZF9 | H517Y | 1 | Missense | ΔZF structure | [^15^](#_ENREF_1) |  |
| ZF10 | Y523* | 1 | Nonsense | Null | [^15^](#_ENREF_1) | >55nt (NMD) |
| ZF10 | Y523fs*8 | 1 | Nonsense | Null | [^42^](#_ENREF_2) | >55nt (NMD) |
| ZF10 | C528* | 1 | Nonsense | Null | [^15^](#_ENREF_1) | >55nt (NMD) |
| ZF11 | R566H | 1 | Missense | ΔZF structure | [^15^](#_ENREF_1) |  |
| ZF11 | R567W | 2 | Missense | ΔZF structure | [^42^](#_ENREF_2) |  |
| ZF11 | N568_splice | 3 | Splice site | Null | [^15^](#_ENREF_1)^,^ [^42^](#_ENREF_2) | >55nt (NMD) |
| ZF11 | P580T | 1 | Missense | ΔZF structure | COSMIC |  |
| C-terminus | E586fs*45 | 1 | Frame shift | Null | [^42^](#_ENREF_2) | >55nt (NMD) |
| C-terminus | E590* | 1 | Nonsense | Null | [^15^](#_ENREF_1) | >55nt (NMD) |
| NLS/Phosphorylation | S604C | 1 | Missense | ? | [^42^](#_ENREF_2) |  |
| NLS/Phosphorylation | S604fs*27 | 1 | Frame shift | Null | [^15^](#_ENREF_1) | >55nt (NMD) |
| C-terminus | S609fs*20 | 1 | Frame shift | Null | [^42^](#_ENREF_2) | >55nt (NMD) |
| C-terminus | E613_splice | 1 | splice site | Null | COSMIC | >55nt (NMD) |
| C-terminus | E616fs*8 | 1 | Frame shift | Null | [^15^](#_ENREF_1) | >55nt (NMD) |
| C-terminus | V630* | 1 | Nonsense | Null | [^42^](#_ENREF_2) | >55nt (NMD) |
| C-terminus | E631* | 1 | Nonsense | Null | [^15^](#_ENREF_1)^,^ [^42^](#_ENREF_2) | >55nt (NMD) |
| C-terminus | I632fs*10 | 1 | Frame shift | Null | [^15^](#_ENREF_1) | >55nt (NMD) |
| C-terminus | P634L | 1 | Missense | ? | [^42^](#_ENREF_2) |  |
| C-terminus | P638L | 1 | Missense | ? | [^15^](#_ENREF_1) |  |
| C-terminus | G657fs*18 | 1 | Frame shift | ? | [^42^](#_ENREF_2) | <55nt |
| C-terminus | R567W | 1 | Point mutation | ? | [^42^](#_ENREF_2) |  |
| C-terminus | E691fs*30 | 2 | Frame shift | ? | [^42^](#_ENREF_2) | In last exon |
| C-terminus | A694V | 1 | Missense | ? | [^42^](#_ENREF_2)^,^ [^47^](#_ENREF_3) |  |
| C-terminus | L722I | 1 | Missense | ? | [^42^](#_ENREF_2)^,^ [^47^](#_ENREF_3) |  |
| C-terminus | *728L | 1 | Loss of stop | ? | ^42^ |  |

| **Supplementary Table 2:** Proportion of missense or inactivating (nonsense and frameshift) mutations in all reads obtained by DNA Seq and RNA Seq spanning the mutation site in human tumour samples (ns P>0.05, * P<0.05, * P<0.01 and *** P<0.001, Fisher’s exact test). | | | | | | | | |
| --- | --- | --- | --- | --- | --- | --- | --- | --- |
| **Mutation  Type** | **CTCF Mutation** | **DNA Seq Read Number** | | | **RNA Seq Reads Number** | | | **Fisher’s exact test** |
|  |  | **WT** | **Mut** | **%Mut** | **WT** | **Mut** | **%Mut** |  |
| Missense | E2G | 47 | 17 | 26.6 | 5 | 3 | 37.5 | ns |
|  | T123A | 151 | 47 | 23.7 | 0 | 4 | 100 | ** |
|  | T207P | 58 | 36 | 38.3 | 0 | 2 | 100 | ns |
|  | H312R | 35 | 18 | 34.0 | 20 | 13 | 39.4 | ns |
|  | Y343H | 134 | 71 | 34.6 | 17 | 10 | 37. | ns |
|  | S354F | 98 | 30 | 23.4 | 5 | 9 | 64.3 | ** |
|  | K365T | 93 | 71 | 43.3 | 0 | 4 | 100 | * |
|  | R377C | 359 | 137 | 27.6 | 25 | 24 | 49.0 | ** |
|  | R377C | 32 | 72 | 69.2 | 2 | 17 | 89.5 | ns |
|  | R377H | 30 | 32 | 51.6 | 5 | 6 | 54.6 | ns |
|  | R377H | 131 | 125 | 48.8 | 6 | 52 | 89.7 | *** |
|  | P378L | 17 | 14 | 45.2 | 5 | 3 | 37.5 | ns |
|  | P378L | 183 | 73 | 28.4 | 7 | 4 | 36.4 | ns |
|  | R515H | 107 | 66 | 37.9 | 35 | 30 | 45.5 | ns |
|  | H517Y | 112 | 32 | 22.1 | 8 | 10 | 55.6 | ** |
|  | R566H | 191 | 166 | 46.4 | 9 | 26 | 74.3 | ** |
| Nonsense | G19fs | 50 | 19 | 38.0 | 7 | 0 | 0 | ns |
|  | Q72* | 107 | 73 | 40.6 | 6 | 12 | 66.7 | * |
|  | E112* | 33 | 44 | 57.1 | 28 | 18 | 39.1 | ns |
|  | E145* | 75 | 29 | 27.9 | 6 | 0 | 0 | ns |
|  | E179* | 135 | 38 | 21.7 | 7 | 0 | 0 | ns |
|  | Q180* | 91 | 53 | 36.8 | 2 | 0 | 0 | ns |
|  | Q186* | 90 | 69 | 43.4 | 5 | 2 | 28.6 | ns |
|  | Y197* | 102 | 59 | 36.7 | 5 | 0 | 0 | ns |
|  | Q198* | 102 | 59 | 36.7 | 5 | 0 | 0 | ns |
|  | I257fs | 134 | 47 | 35.1 | 4 | 0 | 0 | ns |
|  | T318fs | 77 | 19 | 24.1 | 5 | 0 | 0 | ns |
|  | T318fs | 180 | 25 | 13.7 | 30 | 0 | 0 | 0.0519 |
|  | Y358fs | 245 | 49 | 19.8 | 13 | 0 | 0 | ns |
|  | D390fs | 108 | 37 | 32.7 | 16 | 3 | 18.8 | ns |
|  | R448* | 359 | 260 | 42.0 | 16 | 0 | 0 | *** |
|  | R448* | 341 | 54 | 13.7 | 15 | 0 | 0 | ns |
|  | R448* | 202 | 38 | 15.8 | 8 | 0 | 0 | ns |
|  | R448* | 137 | 87 | 38.7 | 5 | 0 | 0 | ns |
|  | R448* | 97 | 16 | 14.2 | 18 | 1 | 5.3 | ns |
|  | Y523* | 84 | 54 | 39.1 | 10 | 1 | 9.1 | 0.0551 |
|  | E590* | 212 | 42 | 16.5 | 12 | 1 | 7.7 | ns |
|  | S604fs | 395 | 133 | 33.3 | 32 | 0 | 0 | *** |
|  | E616fs | 45 | 20 | 44.4 | 4 | 0 | 0 | ns |
|  | E631* | 51 | 19 | 27.1 | 5 | 1 | 16.7 | ns |
|  | I632fs | 80 | 37 | 46.3 | 17 | 0 | 0 | ** |
|  |  |  |  |  |  |  |  |  |

**Supplemenary Figure Legends**

**Supplementary Table 3: Knockdown of CTCF results in gene deregulation in KLE.**

A list of significantly differentially expressed genes comparing KLE expressing shControl and shCTCF following 8 days of Dox treatment.

**Supplementary Figure 1: CTCF haploinsufficiency and survival in endometrial cancer patients.**

Survival analysis of endometrioid endometrial cancer samples for (A) disease-free survival (*CTCF* diploid n=201, *CTCF* mutation n=41, *CTCF* copy loss n=35) and (B) overall survival (*CTCF* diploid n=221, *CTCF* mutation n=44, *CTCF* copy loss n=39). A single sample with both a *CTCF* mutation and a genetic deletion was excluded from analysis. Survival analysis serous endometrial cancer samples for (C) disease-free survival (*CTCF* diploid n=15, *CTCF* copy loss n=28) and (D) overall survival (*CTCF* diploid n=17, *CTCF* copy loss n=34). Survival analyses were compared using the log-rank (Mantel Cox) test. P values were not significant unless indicated.

**Supplementary Figure 2: Image analysis of KLE endometrial spheroids.**

(A) Relative average spheroid area. (B) Relative average spheroid number (C) Relative average intensity of ZO-1 staining and (D) Relative average intensity of F-Actin staining as measured using Volocity 6.3 (Perkin Elmer). ns indicates P>0.05 compared to shCTCF plus Dox , if significant P value is provided. n=4 independent experiments.

**Supplementary Figure 3: CTCF is not deleted specifically in relapse or metastasis.**

A custom Nanostring nCounter Copy Number Variation (CNV) Code Set was designed to span cancer-associated genes in the long arm of chromosome 16 with particular focus on genes within 16q22.1 containing the *CTCF* locus (average of 3 probes, indicated by red border). Chromosome band and gene name are labelled above. Four matched primary and relapse samples and four primary and metastatic samples were analysed. CNV counts were normalised to the average of seven normal or benign endometrium samples which were all considered diploid and any variation depicted on the heatmap. Federation Internationale de Gynecologie et d'Obstetrique (FIGO) tumour grading scores are shown. Endometrial cancer histologies included endometrioid (Endo) and Serous (Ser). Where mixed histologies were seen multiple subtypes are listed. The sites of local relapse or metastasis are listed, and if detected at diagnosis (at diag); (LN) lymph node.
