# Supplementary figures and images for: CTCF genetic alterations in endometrial carcinoma are pro-tumorigenic
Source: Oncogene. 2017 Mar 20;36(29):4100–10. doi: 10.1038/onc.2017.25 (PMC5519450; doi:10.1038/onc.2017.25)

## Endometrioid Endometrial Adenocarcinomas

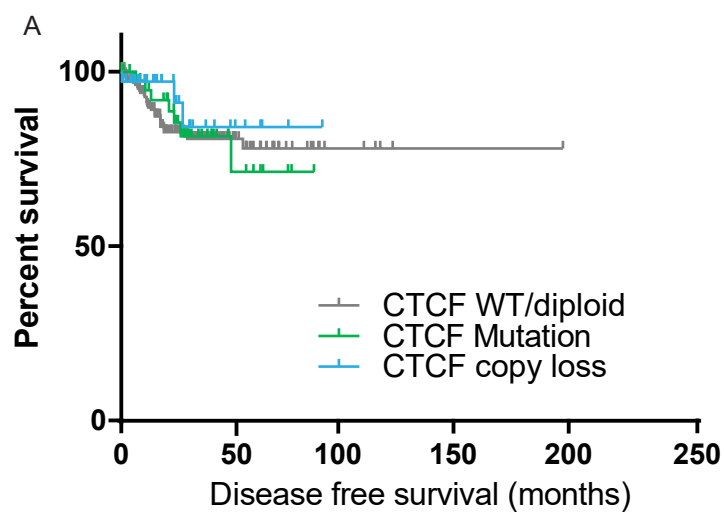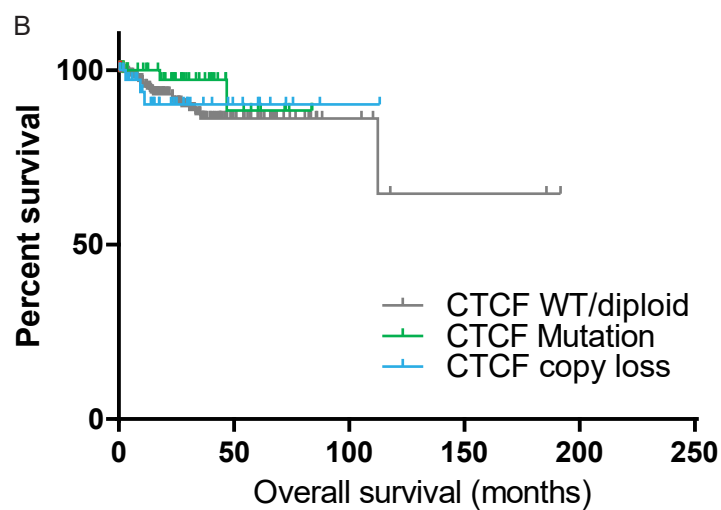

## Serous Endometrial Carcinomas

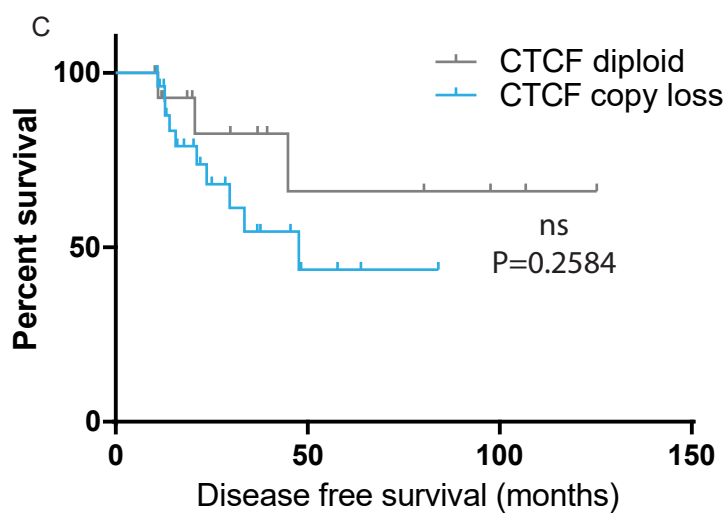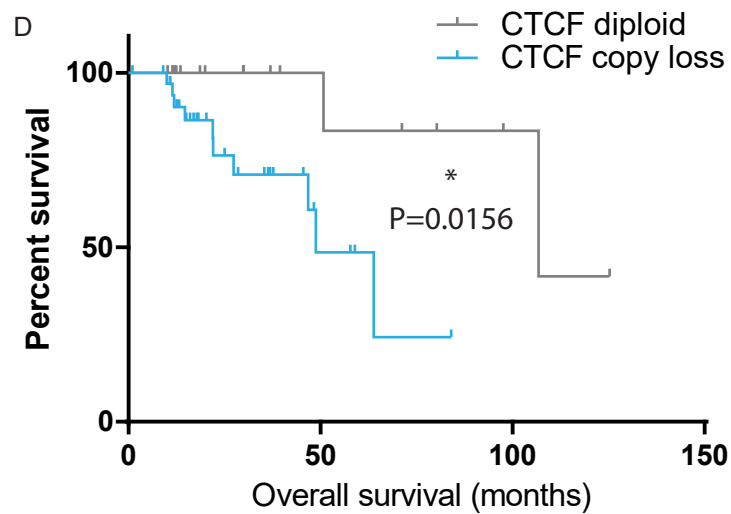

Supplement: Supplementary Information [file onc201725x2.pdf]

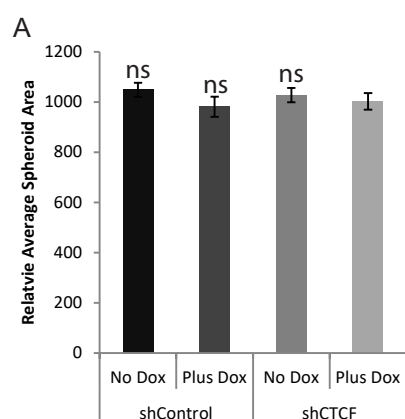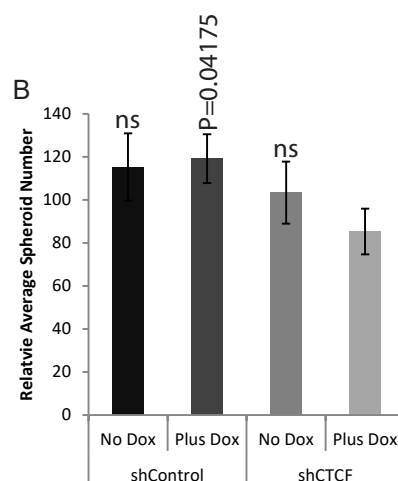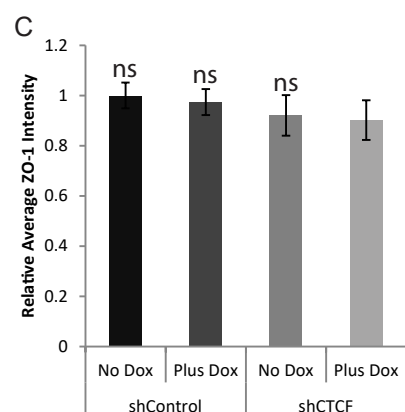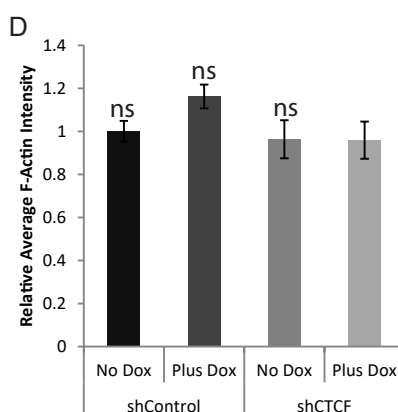

Supplement: Supplementary Information [file onc201725x3.pdf]

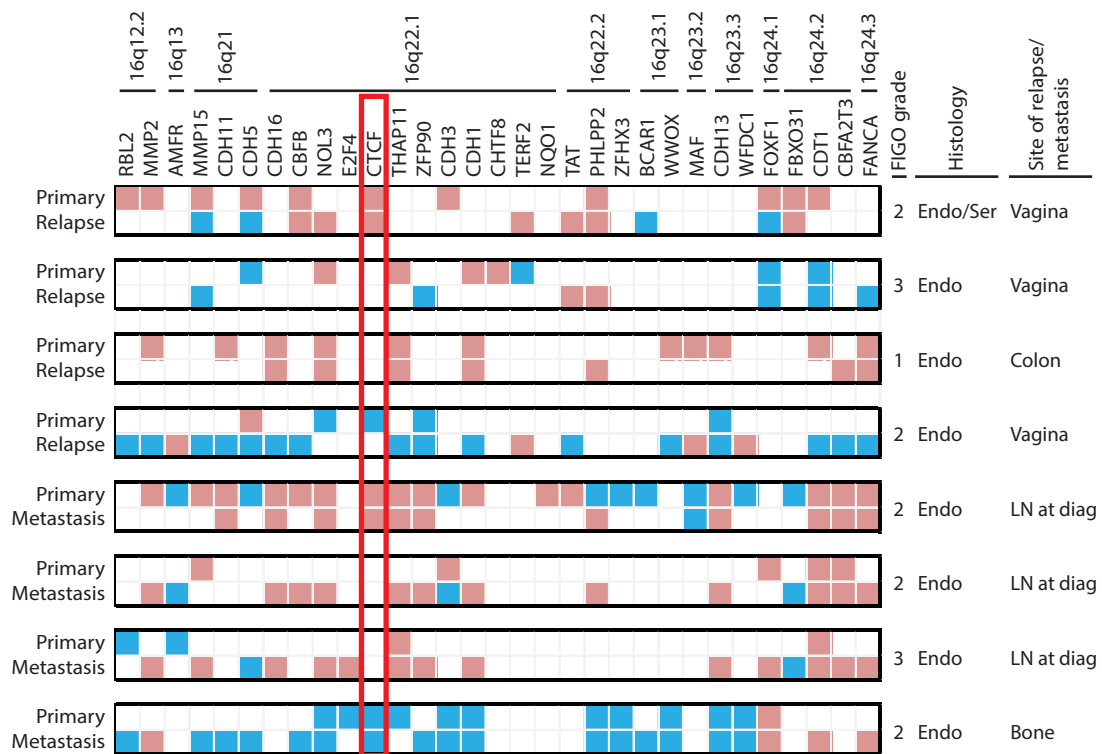

Supplement: Supplementary Information [file onc201725x4.pdf]
